# Training basic numerical skills in children with Down syndrome using the computerized game “The Number Race”

**DOI:** 10.1038/s41598-020-78801-5

**Published:** 2021-01-22

**Authors:** Francesco Sella, Sara Onnivello, Maristella Lunardon, Silvia Lanfranchi, Marco Zorzi

**Affiliations:** 1 Center for Mathematical Cognition, Loughborough University, Loughborough, UK; 2 University of Padova, Padua, Italy; 3 IRCCS San Camillo Hospital, Venice, Italy

**Keywords:** Human behaviour, Neurodevelopmental disorders

## Abstract

Individuals with Down syndrome (DS) present reduced basic numerical skills, which have a negative impact on everyday numeracy and mathematical learning. Here, we evaluated the efficacy of the adaptive (non-commercial) computerized game “The Number Race” in improving basic numerical skills in children with DS. The experimental group (EG; N = 30, *M*_age-in-months_ 118, range 70–149) completed a training playing with “The Number Race”, whereas children in the control group (CG; N = 31, *M*_age-in-months_ 138, range 76–207) worked with software aiming at improving their reading skills. The training lasted 10 weeks with two weekly sessions of 20–30 min each. We assessed both groups’ numerical and reading skills before and immediately after the end of the training, as well as at a 3-months follow-up. We found weak evidence for post-training groups differences in terms of overall numeracy score. However, the EG displayed substantial improvements in specific numerical skills and in mental calculation, which were maintained over time, and no improvement in reading. Conversely, the CG showed improvements in their reading skills as well as in number skills but to a lesser extent compared to the EG. Overall, “The Number Race” appears as a suitable tool to improve some aspects of numeracy in DS.

## Introduction

Numeracy constitutes an essential skill as it has been related to overall academic achievement, financial security, and well-being^1–6^. An adequate numeracy level is also necessary to accomplish those numerical tasks that are part of everyday life, such as calculating bills or managing money proficiently. Everyday math constitutes a crucial goal, especially for individuals with intellectual disabilities^7^. In this regard, training numerical skills becomes relevant in Down syndrome (DS), which is one of the most frequent conditions characterised by intellectual disability^8^.

### Basic numerical skills in Down syndrome

DS results from the trisomy of chromosome 21 and it is characterised by physical abnormalities (e.g., growth delay, flat hypoplastic face with short nose) and intellectual disability^9^. The cognitive profile of DS presents compromised verbal abilities and relatively preserved visuospatial skills^10^.

Individuals with DS display numerical and mathematical skills that are lower compared to typically developing individuals who are matched on chronological age and, in some cases, lower to individuals matched on mental age^11,12^. Several authors have tried to disentangle whether the observed poor math achievement can be fully explained by the lower intelligence or it is the consequence of specific numerical deficits, especially in the basic numerical skills that are supposed to be the building blocks of mathematical learning.

There are two mechanisms responsible for representing small and large non-symbolic numerical quantities, respectively^13,14^. The Object Tracking System is a domain-general system that allows individuals to rapidly and accurately represent small numerical quantities, usually less than 4 items. The fast and accurate enumeration of small sets, known as subitizing^15,16^, is a direct expression of the limited capacity of the OTS, which has been related to visuospatial working memory^17,18^. Large numerical quantities, instead, are processed by the Approximate Number System^13,14,19,20^, whereby each numerosity is represented by a Gaussian distribution of activation on a compressed number line. As a consequence, the numerosities progressively overlap giving rise to a ratio-dependent discrimination performance. That is, the accuracy in discriminating between sets decreases as the ratio between the two numerical quantities gets close to 1 (e.g., easy 8 vs 12; hard: 16 vs 20).

Young children (30-month-old) with DS failed to discriminate between sets with two and three objects, whereas adults with DS (24-year-old) displayed a performance in a numerosity comparison task similar to control individuals^21^. Six-year-old children with DS displayed the expected ratio-dependent effect in a numerosity comparison task as they could discriminate between 8 and 16 dots (1:2 ratio) but failed to discriminate between 8 and 12 (2:3 ratio) as similarly done by controls matched on both chronological and mental age^22^. Similarly, children with DS displayed a performance that decreased as a function of the numerical ratio and was higher for large compared to small numerical quantities^23^. In line with this evidence, two eye-tracking studies showed that children with DS demonstrate looking patterns, when performing a numerosity comparison task, similar to typically developing individuals matched for mental age^24,25^. Conversely, others have found that children with DS displayed a lower ability in comparing numerical quantities compared to children matched on mental age, but not in performing non-symbolic addition^26^. Sella, Lanfranchi, and Zorzi^27^ used a match-to-sample task to explore the ability to discriminate between small and large numerical quantities in children with DS (14-year-old) and controls. Children with DS displayed a lower performance in comparing small numerical quantities (i.e., 2 vs 3, 3 vs 4) compared to children matched on mental and chronological age. The performance in comparing large numerical quantities, instead, was lower only compared to that of chronologically matched individuals but similar to that of mental age controls. Overall, it emerges that individuals with DS display a specific numerical deficit concerning the processing of small numerical quantities. In contrast, the processing of large numerical quantities appears to be in line with mental age^23^. The deficit in processing small numerical quantities might be explained by a limited capacity of the OTS. Accordingly, children with DS show a tracking system whose capacity is limited to one element^28^ and a specific deficit in processing more than one item at a time^29^.

The development of early numerical skills is characterised by a progressive integration between non-symbolic and symbolic representations of numbers^30^. Initially, young children can discriminate between small and large non-symbolic numerical quantities^31–33^. Around the age of 2, children learn how to recite the number sequence in its stable order. However, it takes almost two years of age to learn and master counting by repeatedly associating number words with the respective external numerical quantities^34,35^. Thereafter, children understand the magnitude relation between numerical symbols (i.e., number words and Arabic digits^36,37^). The ability to compare non-symbolic and symbolic numerical quantities has been repeatedly associated with mathematical achievement^38^. The more reliable association is between symbolic number comparison and arithmetic fluency, as magnitude understanding supports the choice of an efficient calculation strategy (e.g., counting from larger^39^).

On the one hand, it has been suggested that children with DS perform counting as a rote behaviour without having a deep understanding of the cardinality principle (i.e., understanding that the last pronounced number word corresponds to the cardinality of the set^34,40^), which in turn prevents them from developing more advanced numerical skills^41^. On the other hand, some authors have argued that children with DS master the counting principles^42,43^, but have a reduced number word sequence^44,45^ and they are slower when counting items, although comparable to typically developing children matched on mental age^27^. For instance, Sella, Lanfranchi and Zorzi^27^ asked children with DS and controls matched on mental and chronological age to perform a match to sample task, in which children had to verify whether the numerosity of a given visual set matched a previously presented Arabic numeral. Children with DS displayed an accuracy slightly lower but mostly comparable to MA children, even though their response times were slower. Overall, it seems that children with DS can understand the counting principles, but their poor counting performance might be related to limited vocabulary capacity and deficit in implementing counting behaviour^46^.

Children with DS also display a poor performance when translating numbers into a spatial position as assessed by the number line task^47–49^, whereby participants place several consecutively presented target numbers on a visual horizontal line entailing a numerical interval. For instance, participants mark the location of the target number 23 on a line with the number 0 on the left-end side and the number 100 on the right-end side. It has been repeatedly observed that children display a shift from a biased (log-like) to an accurate (linear) positioning with increased numerical knowledge and experience with the proposed numerical interval. Accordingly, preschool children display a linear positioning in the 1–10 interval but a biased one in the 0–100 interval^50^, whereas older children display a linear positioning also in the 0–100 interval^47^. Children with DS (14 years-old) displayed a linear positioning in the interval 1–10, in line with the performance of the control group matched on mental age, even though their precision in positioning numbers was lower compared to typically developing children matched on chronological age. In the interval 0–100, both DS and mental age-matched controls displayed a biased (log-like) positioning, whereas the positioning of the chronological age-matched group was linear^51^.

The poor performance in the number line task could be related to a lack of arithmetic strategies when solving the task, such as using the midpoint of the line as an anchoring point^52–56^. Several studies have shown that individuals with DS have reduced arithmetic skills as measured on standardized tests. Brigstocke et al.^11^ reported that only slightly more than half of the children with DS in their sample could complete a battery to assess basic numerical skills, including arithmetic, and their scores were yet extremely low. Similarly, it has been found that only half of the adolescents with DS can perform simple additions^57^ and adults with DS can recognise numbers and count, but their arithmetic skills are essentially absent^58^.

In summary, individuals with DS have reduced ability to process small numerical quantities compared to controls matched on mental age, whereas the ability to discriminate large numerical quantities is in line with mental age. Children with DS seem to master the counting principles, even though they are slower when enumerating items and more prone to commit errors. The accuracy in positioning numbers on the visual line is line with mental age whereas arithmetic skills are severely reduced. It is worth highlighting that these specific numerical deficits might be the byproduct of domain-general factors. Accordingly, the deficit in comparing small numerical quantities can be related to poor visuospatial working memory capacity. Similarly, the counting deficit in DS could be attributed to poor knowledge of the number sequence, which in turn, depends on verbal memory.

### The present study

Most of the interventions to improve numerical skills in DS have focussed on early numerical skills. Previous studies have reported preliminary evidence on the effectiveness of programmes to improve basic numerical skills^59,60^, non-symbolic number comparison^61^, counting^62–65^, arithmetic^66,67^, conservation of numerical quantities^68,69^, and fractions^70^. However, the research in this area is still scarce and presents relevant methodological limitations^71^. Accordingly, most of the training studies lack an active control group and/or follow-up assessment, which makes it difficult to evaluate the efficacy of the training and its long-lasting effects.

Here, we evaluate the effectiveness of the adaptive computerized game “The Number Race”^72–74^ in improving basic numerical skills in children with DS. The game (which is freely available for non-commercial use in multiple language versions) targets those numerical skills that are usually acquired in the preschool time period. The structure of the game is based on four principles: enhancing number sense; cementing the links between representations of number; conceptualizing and automatizing arithmetic; maximizing motivation. Players compete against the software in a numerical comparison task, choosing the larger between two numerical quantities ranging from 1 to 9, which may be sets of dots, digits, or the results of sums or subtractions. An adaptive algorithm modulates the presentation time of the to-be-compared numerical quantities, the size of the dots, or the numerical distance to keep the difficulty of the game at a challenging level, thus working on the *zone of proximal learning*^75^. The training on the comparison of small and large non-symbolic and symbolic numerical quantities and basic arithmetic makes the software an appropriate tool as children with DS display difficulties in such abilities. The player chooses the larger quantity, so the other quantity is given to the opponent (the software). Afterwards, a board with 40 cells (4 × 10) is presented in a different screen, and players can move their characters as many steps as the numerical quantity they chose in the comparison. The request to move the characters on the game board improves children’s ability to positioning numbers on a spatial layout^73,76,77^, which is another impaired ability in children with DS. The race ends when one of the characters reaches the end of the board. Verbal and acoustic feedback is continuously provided to foster motivation. The software has already provided preliminary, but promising, results on its efficacy of improving basic numerical skills in young children and in children with math difficulties^73,78–81^, even though this research presents methodological limitations and more studies are needed^82^.

Here, we present the results of an intervention study, whereby children with DS in the experimental group (EG) played with “The Number Race” whereas children in the control group (CG) worked with software aiming at improving their reading skills. The use of an active control group was designed to ensure a stringent evaluation of the numerical training effects. Though assessing numerical improvements was the primary aim of the present study, we also evaluated whether the reading training may improve literacy in DS. We assessed both groups’ numerical and reading skills at pre-test, post-test, immediately after the end of the training, and at follow-up, after 3 months. We expected the EG to show relevant improvements in their numerical, but not reading, skills from pre-test to post-test compared to the CG. Such improvement might be still evident at follow-up, months after the end of the training. Conversely, we expected the CG group to show a larger improvement in reading skills compared to the EG group.

## Methods

### Participants

Forty-one children with Down Syndrome (DS) from north-eastern Italy took part in the study, after obtaining informed consent from parents and verbal consent from participants. We recruited participants from local associations, which offer support to families of children with intellectual disabilities. We evaluated at pre-test all underage children willing to take part as long as their parents considered them capable of completing the training under the supervision of the experimenter. However, we excluded two participants that at pre-test were not able to complete the standardized numeracy battery or had serious behavioural issues during the testing session. We allocated 20 participants (12 boys, age range in months = 70–149) to the Experimental Group (EG), that played with the Italian version of “The Number Race”^72,74^, and 21 participants (14 boys, age range in months = 76–207) to the active Control Group (CG), that practised with the software “Fondiamoleletterine”^73^ (it translates to “Let’s Fuse Letters”) or “Lettura di base 3”^83^ (it translates to “Basic Reading 3”), which aim to improve reading skills. Among children in the CG, 12 played only with “Fondiamoleletterine”, 6 only with “Lettura di Base 3” and 3 with both. This subdivision was done considering the child’s reading ability. The two groups were matched on age, fluid intelligence (Raven’s Colored Matrices^84^), and receptive vocabulary (Peabody Picture Vocabulary Test-Revised; PPVT-R^85^). Children were assessed on these tasks by the experimenter in one of the testing sessions before the beginning of the training. Participants’ characteristics are reported in Table 1. The age equivalent scores of receptive vocabulary (age-in-months = 51.90) and fluid intelligence (age-in-months = 65.80) were in line with those expected from a sample of children with DS between 10–11 years of age. Children with DS also displayed the typical cognitive profile with better non-verbal than verbal age equivalent scores (i.e., vocabulary).

**Table 1:** Experimental Group (EG) and Control group (CG) characteristics.

Measure	EG n = 20	CG n = 21	*t*(38)	*p*	BF_10_
	*M *(*SD*)	*M *(*SD*)			
Chronological age (in months)	118.10 (24.59)	137.62 (38.65)	− 1.912	0.062	1.284
Receptive vocabulary (PPVT-R)	55.90 (21.09)	48.10 (17.38)	− 1.296	0.203	0.595
Intelligence (CPM)	68.50 (18.61)	63.24 (17.95)	− 0.922	0.362	0.429

Mean (SD).

### Procedure

We assigned participants to the EG or CG alternatively, that is, the first participant was assigned to the EG, the second to the CG, the third to the EG and so on. The parents of four children explicitly demanded that their children were delivered the numerical training; therefore, these participants were allocated in the EG (this deviation was deemed as acceptable in light of the difficulty in recruitment). We assigned the last four participants to the CG to obtain a balanced sample size in the two groups. After participants were allocated to the experimental or control group, we assessed their cognitive skills in three testing phases (i.e., pre-test, post-test, and follow-up), each composed of four 45-min separate sessions on different days. The pre-test and post-test phases were completed respectively before and after the training, whereas participants completed the last follow-up session three months after the end of the training. The testing and training sessions were run in a comfortable and quiet room, individually, at home, at the clinical centre, or at school premises according to participants’ availability. Trained graduate students, who were not blind to group allocation, completed the assessments at pre-test, post-test, and follow-up and supervised children during training. We recruited participants between April 2016 and August 2019 considering the research project resources in terms of staff and working hours. The present study was approved by the Ethics Committee for Psychological Research of the University of Padova and it was performed in accordance with the Declaration of Helsinki. The trial was not pre-registered and there were no substantial changes in the methods or outcome variables after the trial began.

### Tasks

We reported the test–retest reliability for the standardised tests whereas for the other tasks we calculated the split-half reliability by correlating the scores, separately for each session, between odd and even trials, and then applying the Spearman-Brown formula (i.e., [2 × *r*]/[1 + *r*]).

#### Numerical intelligence battery (BIN: Batteria Intelligenza Numerica)

The BIN^86^ is a standardized numeracy test designed for preschool-level children and composed of four subscales: lexical, semantic, counting, and pre-syntactic. The battery demonstrated good psychometric properties, with all subscales presenting high reliability (Lexical subscale: *r* = 0.89, Semantic subscale: *r* = 0.69, Counting subscale: *r* = 0.74, Pre-syntactic subscale: *r* = 0.79). The lexical subscale assesses the ability to read and write Arabic numbers as well as the ability to connect number-words to the corresponding digits. The semantic subscale measures the ability to compare numerical quantities (i.e., dots and Arabic digits). The counting subscale assesses the ability to recite the number–words sequence forward and backward as well as the knowledge of the order of Arabic digits from 1 to 5. The pre-syntactical scale evaluates the ability to link numbers to sets of dots and to order objects based on their size. We used the sum of the four subscales as an index of basic numerical abilities.

#### Number words comparison

In this task, children were asked to indicate the larger between two number words. The experimenter said: “*Which is ‘more’ between* x *candy/ies and* y *candy/ies?*”, where x and y were number words ranging from 1 to 9. The comparison were: 4vs2, 2vs7, 3vs8, 2vs1, 8vs7, 5vs4, 3vs6, 7vs6, 1vs5, 9vs3, 1vs4. We calculated the percentage of errors as the outcome measure. For this task, we only collected the total number of correct responses, thereby preventing us from calculating the split-half reliability. However, the same task showed split-half reliability of 0.82 when calculated on data from a previous study with preschool children^36^.

#### Mental calculation task

The experimenter sequentially read aloud eight-teen arithmetic problems (8 additions and 10 subtractions), and the child had to respond as fast as they could. Children could count on their fingers to calculate the answer. We calculated the percentage of correct responses. The split-half reliability was 0.95 at pre-test, 0.97 at post-test, and 0.97 at follow-up.

#### Number-to-position task (NTP^49,50,87^)

Children were presented with a 20-cm line on a white landscape sheet. The left end of the line was labelled with the number 1 and the right end side was labelled either with 10 or 20. The target number to be positioned was shown on the left upper corner of the sheet. For each interval, there were eight randomly presented target numbers (i.e., 2, 3, 4, 5, 6, 7, 8, 9 for the 1–10 interval; 2, 4, 6, 7, 13, 15, 16, 18 for the 1–20 interval). Every trial, a new number line was presented with a different target number to be placed. The experimenter said: “Now we are going to play a game with number lines. You can see that this line goes from 1 to 10 (or 20). I will tell you a number and you have to indicate which is the place of this number on the line, as precise as you can.” The instructions were repeated as many times as needed, but no feedback was given. As training trials, children had to place 1 and 10 in the 1–10 line, whereas in the 1–20 line the training trials were 1 and 20. The experimenter named the target numbers every trial. Children drew a vertical mark on the line where they thought the target number should be placed. Some children had difficulties in holding the pencil, so they were asked to point with their finger the position of the target number on the line and the experimenter made the mark. An index of accuracy on this task was obtained by computing the individual percentage of absolute error ([[|Estimate-Target Number|]/Numerical Interval]×100). The split-half reliability for the range 0–10 was 0.78 at pre-test, 0.87 at post-test and 0.87 at follow-up; for the range 0–20, it was 0.77 at pre-test, 0.84 at post-test and 0.87 at follow-up.

#### Match-to-sample task

A sample set of white dots on a gray background appeared in the centre of the computer screen for 300 ms. Then, another set of black dots appeared on the screen and the child indicated whether the numerosity of the set was the same or different compared to the sample set (i.e., white dots). The numerosity of the target set (i.e., black dots) could be the same or minus/plus one compared to the sample set. There was no time restriction to provide the answer as the target set remained on the screen until the child responded. Size and spatial arrangement of the dots changed in each trial to prevent children based their response on non-numerical visual cues. There were 90 experimental trials: 12 trials for each sample numerosity from 2 to 7, and 9 trials for 1 and 8. Before starting the task, participants completed ten training trials, whereby the presentation of the sample set was longer and decreased trial by trial, to help the child familiarise with the task. We calculated the percentage of correct responses for each participant. The split-half reliability was 0.58 at pre-test, 0.56 at post-test, and 0.62 at follow-up.

#### Number naming

The child read aloud the Arabic number presented in the middle of the computer screen. We showed all the numbers from 0 to 20 in random order and calculated the percentage of correct responses. The split-half reliability was 0.97 at pre-test, 0.96 at post-test, and 0.97 at follow-up.

#### Counting

A set of objects (i.e., bananas or apples) appeared on the screen and the child counted it as fast as possible. There were 2 trials for each of the following target numerosities: 1, 2, 3, 4, 5, 8, 10. We calculated the percentage of correct responses. The split-half reliability was 0.80 at pre-test, 0.84 at post-test, and 0.78 at follow-up.

#### Digit comparison

Children indicated the larger between two digits, ranging from 1 to 9, that were presented on the left and right side of the computer screen respectively. There were 72 trials presenting all the possible comparisons of digits between 1 and 9, each comparison repeated twice, once with the larger digit on the right side and once with the larger digit on the left side of the screen. The split-half reliability was 0.93 at pre-test, 0.93 at post-test, and 0.91 at follow-up.

#### Letter recognition task^88^

The experimenter read aloud a letter in a triplet of letters and the child indicated the corresponding letter. There were 21 triplets of letters and children obtained a point for each correct recognition. We calculated the percentage of errors for each child. The split-half reliability was 0.91 at pre-test, 0.86 at post-test, and 0.85 at follow-up.

#### Syllable reading^89^

Children read aloud a series of syllables (a matrix of 10 × 10), in order, from left to right, as fast as possible. The number of errors was calculated for each child. The split-half reliability was 0.997 at pre-test, 0.996 at post-test and 0.997 at follow-up.

#### *Word and pseudoword reading*^90^

Children read aloud four lists of 28 words and three lists of 16 pseudo-words. The lists were presented one at the time. We calculated the total number of errors for each child. Test–retest reliability was *r* = 0.56.

The selected numerical tasks measure those abilities that are the target of the Number Race. Accordingly, the Number Race repeatedly asks children to compare non-symbolic and symbolic numerical quantities as measured in the match-to-sample, number words comparison, and Arabic digit comparison tasks. Some children might count the dots in each set, thereby using serial counting, which we assessed in the counting task. In the advanced stages of the game, the to-be-compared numerical quantities are the results of additions and subtractions, which were tested in the mental calculation task. Moreover, the software always reads aloud the Arabic digits so children can improve the connection between visual and verbal representation of numbers, which we measured using the naming task. The Number Race asks children to move the game characters on a linear board, thereby improving the association between number and space, which was evaluated using the number line tasks. It is worth noting that the administered tasks were structurally similar to the component tasks that form the Number Race game. A notable exception is the number line task(s) because children were not required to mark the spatial position of target numbers in the Number Race. In this vein, the number line tasks could be considered as near transfer tasks. Finally, the BIN is a standardised battery assessing different aspects of numerical knowledge in preschool children, which is the corresponding mental age of the participants with DS. Similarly, we selected the letter recognition, syllables reading, and word and pseudoword reading task to assess the effectiveness of the control (reading) intervention.

### Training

Both the experimental and the active control group completed 20 training sessions across ten weeks, with two weekly sessions of 20–30 min each. The experimenter supervised participants during the training sessions and continuously provided feedback to support participants’ engagement with the training. Participants were encouraged to engage with the training activity for at least 20 min, but no more than 30 min. The training was delivered on the experimenter’s laptop and all children completed the planned number of sessions.

Children in the experimental group played with the Italian version of “The Number Race” game^72–74^. Children in the active control group underwent an intervention based on “Fondiamoleletterine”^91^ or “Lettura di base 3”^83^, according to their age and level. The former is a software aimed to support the early steps of reading acquisition, training phoneme blending. Moreover, it has been shown to be effective in children with DS, in particular, leading to improvements in decoding of syllables and words and repetition of auditory stimuli^92^. It is composed of seven levels of increasing difficulty: in the first one, letters are associated by shape and sound to a figure (to facilitate memorization), while in the following levels, phoneme blending is trained, starting from syllables of increasing difficulty (consonant + vowel at the beginning, three-letter syllables later), to disyllabic words. The child can then listen to the syllable/word reproduced by the software. The latter intervention improved word reading. The game is composed of different kinds of activities, where the child is required to read words or brief texts, for example, phonemic and syllabic inference, word-picture association, vertical word reading. The software gave feedback and reinforcements to the participant and all the activities were set in a playful environment to make exercises pleasant and motivating. The syllables and words utilized in these training sessions were not the same adopted in the testing sessions.

## Results

We analysed the effect of the training by running mixed ANOVAs for each task with Session (Pre-test vs. Post-test vs. Follow-up) as a within-subjects factor and Group (EG vs. CG) as a between-subjects factor. When the assumption of Sphericity was violated, the Greenhouse–Geisser adjustment was applied to *p*-values (reported as *p*_[gg]_). Post-hoc t-tests were two-tailed and the *p*-values were corrected for multiple comparisons analysis using the Bonferroni method (i.e., alpha value divided by the number of comparisons). Hedges’ g was calculated to determine the magnitude of the difference between the groups at each session. We also reported Bayes factors (BF_10_) expressing the probability of the data given H1 relative to H0 (i.e., values larger than 1 are in favour of H1 whereas values smaller than 1 are in favour of H0^93,94^). We reported the Bayes factors (BF) as the ratio of BFs_10_ between compared models. If the ratio between BF_10_ of model A and BF_10_ of model B is larger than 1, then there is evidence for model A. Conversely, if the ratio is smaller than one, there is evidence for model B. We reported the scores in the administered tasks across sessions for the two groups in Table 2. The zero-order correlations between all pre-test scores are reported in Table S1 in the Supplementary Information.

**Table 2:** Mean scores (*SD*) [range] at pre-test, post-test, and follow-up separately for the EG and CG groups.

Measures	EG (N = 20^a^)			CG (N = 21^a^)		
	Pre-test	Post-test	Follow-up	Pre-test	Post-test	Follow-up
BIN (correct responses) Max score = 106	71.70 (23.25) [31–105]	84.75 (17.95) [53–106]	85.55 (17.96) [56–105]	72.00 (24.62) [24–105]	77.33 (22.24) [31–106]	77.57 (21.91) [34–106]
Number comparison (% of errors)	29.09 (18.56) [0–63.64]	14.55 (15.15) [0–45.45]	14.09 (14.59) [0–45.45]	29.87 (21.92) [0–81.82]	25.54 (18.32) [0–54.55]	30.30 (19.78) [0–63.64]
Mental calculation (% of correct responses)	23.81 (32.79) [0–94.44]	47.22 (43.19) [0–100]	50.00 (42.98) [0–100]	29.63 (37.53) [0–100]	38.27 (44.81) [0–100]	32.10 (42.55) [0–100]
NTP 0–10 (PAE)	20.43 (9.46) [7.53–44.44]	17.13 (10.55) [4.33–42.43]	15.53 (11.16) [3.06–50.88]	22.17 (11.56) [3.53–44.44]	20.64 (13.83) [5.79–50]	23.64 (15.85) [4.60–54.17]
NTP 0–20 (PAE)	22.33 (11.48) [2.44–48.19]	16.56 (11.09) [3.28–44.21]	14.65 (9.76) [5.88–47.64]	22.10 (13.06) [2.44–49.10]	24.51 (16.97) [3.48–59.37]	27.45 (18.43) [4.60–59.21]
Match-to-sample (% correct)	52.54 (7.98) [38.89–64.44]	61.43 (4.54) [53.33–68.89]	61.98 (9.03) [43.33–76.67]	57.89 (8.05) [46.67–70.00]	58.00 (8.95) [45.56–71.11]	59.00 (10.55) [45.56–76.67]
Naming (% correct)	67.35 (32.93) [23.81–100]	69.73 (26.06) [38.10–100]	68.03 (26.90) [38.10–100]	75.00 (28.92) [19.05–100]	73.81 (30.39) [14.29–100]	72.22 (30.70) [19.05–100]
Counting (% correct)	76.42 (19.19) [28.57–93.33]	84.69 (13.39) [57.14–100]	84.18 (10.55) [71.43–100]	73.81 (26.95) [28.57–100]	76.59 (28.76) [7.14–100]	78.18 (23.17) [21.43–100]
Digit comparison (% correct)	65.48 (17.81) [40.28–98.61]	79.76 (13.88) [52.78–98.61]	78.97 (12.05) [65.28–100]	67.59 (21.04) [45.83–100]	73.38 (20.48) [45.83–100]	73.61 (17.93) [44.44–100]
Letter recognition (% of errors)	15.24 (18.25) [0–71.43]	10.71 (15.28) [0–57.14]	9.76 (13.77) [0–47.62]	15.64 (23.24) [0–80.95]	6.12 (11.87) [0–38.10]	3.85 (9.60) [0–42.86]
Syllable reading (errors) Max score = 100	46.50 (42.48) [0–100]	46.70 (42.79) [0–100]	48.55 (43.37) [0–100]	59.86 (44.08) [6–100]	43.67 (40.11) [2–100]	41.24 (39.02) [1–100]
Word reading (errors) Max score = 112	64.60 (50.33) [0–112]	63.20 (50.99) [0–112]	64.25 (50.73) [0–112]	74.48 (47.54) [8–112]	63.00 (49.77) [5–112]	62.10 (50.89) [3–112]
Pseudoword reading (errors) Max score = 48	31.60 (17.81) [6–48]	30.55 (18.77) [2–48]	31.25 (18.76) [0–48]	34.95 (16.75) [10–48]	31.19 (20.85) [4–48]	30.62 (21.52) [2–48]

^a^Due to a computer failure at follow-up, the sample size was reduced for the following tasks: mental calculation, naming, counting and digit comparison (N_EG_ = 14; N_CG_ = 18) and Match-to-Sample (N_EG_ = 14; N_CG_ = 10).

In Table 3, we reported the results of mixed ANOVAs. The contrasts between and within groups were reported only when the interaction between Session and Group was significant. According to Bonferroni correction, we adjusted the alpha levels to 0.016 (i.e., 0.05/3) for comparisons between groups in each one of the three sessions, and to 0.008 (i.e., 0.05/6) for comparisons between pre-test and post-test, pre-test and follow-up, and post-test and follow-up within the two groups. Due to a computer failure, results at follow-up in mental calculation, naming, counting and digit comparison tasks were missing for 6 children of EG and 3 of CG, and in the match-to-sample results were missing for 6 children of EG and 11 of CG.

**Table 3:** Statistical results of mixed ANOVAs and post-hoc t-tests.

Task	Main effect of session	Main effect of group	Interaction session × group	t-test between groups			t-test within groups					
				Pre-test	Post-test	Follow-up	EG			CG		
							Pre-test vs post-test	Pre-test vs follow-up	Post-test vs follow-up	Pre-test vs post-test	Pre-test vs follow-up	Post-test vs follow-up
BIN	*F*(1.62, 63.18) = 38.93 *p*_[gg]_ < .001 η_p_^2^ = 0.50 BF_10_ = 2.17 × 10^8^	*F*(1, 39) = 0.59 *p* = 0.45 η_p_^2^ = 0.02 BF_10_ = 0.65	*F*(1.62, 63.18) = 6.98 *p*_[gg]_ = 0.003, η_p_^2^ = 0.15 BF_10_ = 3.007 × 10^9^	*t*(39) = 0.04 *p* = 0.97 *g* = 0.01 BF_10_ = 0.31	*t*(39) = − 1.72 *p* = 0.25 *g* = − 0.36 BF_10_ = 0.53	*t*(39) = − 1.27 *p* = 0.21 *g* = − 0.39 BF_10_ = 0.58	*t*(19) = 5.80 *p* < .001 *g* = 0.62 BF_10_ = 1634.83	*t*(19) = 5.65 *p* < .001 *g* = 0.65 BF_10_ = 1223.12	*t*(19) = 0.81 *p* = 0.43 *g* = 0.04 BF_10_ = 0.31	*t*(20) = 3.91 *p* < .001 *g* = 0.22 BF_10_ = 40.67	*t*(20) = 3.55 *p* = .002 *g* = 0.23 BF_10_ = 19.58	*t*(20) = 0.16 *p* = 0.88 *g* = 0.01 BF_10_ = 0.23
	Group + Session: BF_10_ = 1.55 × 10^8^											
Number comparison	*F*(1.58, 61.62) = 10.93 *p*_[gg]_ < 0.001 η_p_^2^ = 0.22 BF_10_ = 99.84	*F*(1,39) = 3.27 p = 0.08, η_p_^2^ = 0.08 BF_10_ = 1.24	*F*(1.58, 61.62) = 6.88 *p*_[gg]_ = 0.004 η_p_^2^ = 0.15 BF_10_ = 2351.23	*t*(39) = 0.12 *p* = 0.90 *g* = 0.04 BF_10_ = 0.31	*t*(39) = 2.09 *p* = 0.04 *g* = 0.64 BF_10_ = 1.66	*t*(39) = 2.97 *p* = 0.005 *g* = 0*.*91 BF_10_ = 8.48	*t*(19) = − 5.00 *p* < 0.001 *g* = − 0.84 BF_10_ = 346.38	*t*(19)= − 4.93 *p* < 0.001 *g* = − 0.88 BF_10_ = 297.47	*t*(19) = − 0.29 *p* => 0.008 *g* = − 0.03 BF_10_ = 0.24	*p* > 0.008 *g* = − 0.21 BF_10_ = 0.51	*p* > 0.008 *g* = 0.02 BF_10_ = 0.23	*p* > 0.008 *g* = 0.25 BF_10_ = 0.90
	Group + Session: BF_10_ = 126.79											
Mental calculation	*F*(1.5, 45) = 10.94 *p*_[gg]_ < 0.001 η_p_^2^ = 0.27 BF_10_ = 47.97	*F*(1,30) = 0.25 *p* = 0.62 η_p_^2^ = 0.01 BF_10_ = 0.59	*F*(1.5, 45) = 5.06 *p*_[gg]_ = 0.02, η_p_^2^ = 0.14 BF_10_ = 135.38	*t*(30) = 0.45 *p* = 0.65 *g* = 0.16 BF_10_ = 0.37	*t*(30) = − 0.57 *p* = 0.57 *g* = − 0.20 BF_10_ = 0.38	*t*(30) = − 1.18 *p* = 0.25 *g* = − 0.41 BF_10_ = 0.57	*p* > 0.008 *g* = 0.59 BF_10_ = 3.44	*p* > 0.008 *g* = 0.67 BF_10_ = 4.72	*p* > 0.008 *g* = 0.06 BF_10_ = 0.32	*p* > 0.008 *g* = 0.20 BF_10_ = 2.03	*p* > 0.008 *g* = 0.06 BF_10_ = 0.41	*p* > 0.008 *g* = − 0.14 BF_10_ = 1.83
	Group + Session: BF_10_ = 29.37											
NTP 1–10	*F*(2,78) = 0.849 *p* = 0.43 η_p_^2^ = 0.02 BF_10_ = 0.15	*F*(1,39) = 2.00 *p* = 0.17 η_p_^2^ = 0.05 BF_10_ = 0.73	*F*(2,78) = 1.48 *p* = 0.23 η_p_^2^ = 0.04 BF_10_ = 0.05									
	Group + Session: BF_10_ = 0.111											
NTP 1–20	*F*(2,78) = 0.51 *p* = 0.61 η_p_^2^ = 0.01 BF_10_ = 0.11	*F*(1,39) = 3.12 *p* = 0.09 η_p_^2^ = 0.07 BF_10_ = 1.21	*F*(2,78) = 7.43 *p* = 0.001 η_p_^2^ = 0.16 BF_10_ = 4.07	*t*(39) = − 0.06 *p* = 0.95 *g* = − 0.02 BF_10_ = 0.31	*t*(39) = 1.77 *p* = 0.09 *g* = 0.54 BF_10_ = 1.04	*t*(39) = 2.76 *p* = 0.009 *g* = 0.84 BF_10_ = 5.46	*p* > 0.008 *g* = − 0.50 BF_10_ = 1.85	*p* > 0.008 *g* = − 0.71 BF_10_ = 2.76	*p* > 0.008 *g* = − 0.18 BF_10_ = 0.29	*p* > 0.008 *g* = 0.16 BF_10_ = 0.41	*p* > 0.008 *g* = 0.33 BF_10_ = 1.87	*p* > 0.008 *g* = 0.16 BF_10_ = 0.83
	Group + Session: BF_10_ = 0.13											
Match-to-Sample	*F*(2,44) = 4.77 *p* = 0.01 η_p_^2^ = 0.18) BF_10_ = 8.48	*F*(1,22) = 0.02 *p* = 0.90 η_p_^2^ = 0.001 BF_10_ = 0.35	*F*(2, 44) = 6.98 *p* = 0.04, η_p_^2^ = 0.14 BF_10_ = 8.07	*t*(22) = 1.61 *p* = 0.12 *g* = 0.64 BF_10_ = 0.94	*t*(22) = − 1.24 *p* = 0.23 *g* = − 0.49 BF_10_ = 0.65	*t*(22) = − 0.74 *p* = 0.46 *g* = − 0.30 BF_10_ = 0.46	*t*(13) = 4.00 *p* = 0.001 *g* = 1.14 BF_10_ = 33.66	*t*(13) = 3.26 *p* = 0.005 *g* = 0.97 BF_10_ = 9.31	*t*(13) = 0.22 *p* > 0.008 *g* = − 0.05 BF_10_ = 0.26	*p* > 0.008 *g* = 0.12 BF_10_ = 0.35	*p* > 0.008 *g* = 0.08 BF_10_ = 0.35	*p* > 0.008 *g* = 0.18 BF_10_ = 0.40
	Group + Session: BF_10_ = 3.12											
Naming	*F*(1.5, 45) = 0.35, *p*_[gg]_ = 0.64, η_p_^2^ = 0.01 BF_10_ = 0.13	*F*(1,30) = 0.27, *p* = 0.61, η_p_^2^ = 0.009 BF_10_ = 0.71	*F*(1.5, 45) = 0.53, *p*_[gg]_ = 0.54, η_p_^2^ = 0.02 BF_10_ = 0.02									
	Group + Session: BF_10_ = 0.09											
Counting	*F*(2,60) = 2.61 *p* = 0.08 η_p_^2^ = 0.08 BF_10_ = 0.65	*F*(1,30) = 0.61 *p* = 0.44 η_p_^2^ = 0.02 BF_10_ = 0.57	*F*(2,60) = 0.45 *p* = 0.64 η_p_^2^ = 0.02 BF_10_ = 0.08									
	Group + Session: BF_10_ = 0.37											
Digit comparison	*F*(2,60) = 17.27 *p* < 0.001 η_p_^2^ = 0.37 BF_10_ = 3189.184	*F*(1,30) = 0.29 *p* = 0.59 η_p_^2^ = 0.01 BF_10_ = 0.53	*F(*2,60) = 2.84 *p* = 0.07 η_p_^2^ = 0.09 BF_10_ = 1865.37									
	Group + Session: BF_10_ = 1836.19											
Letter recognition	*F*(1.32, 51.48) = 11.74 *p*_[gg]_ < 0.001 η_p_^2^ = 0.23 BF_10_ = 617.40	*F*(1,39) = 0.56 *p* = 0.46 η_p_^2^ = 0.01 BF_10_ = 0.49	*F*(1.32,51.48) = 1.55 *p*_[gg]_ = 0.22 η_p_^2^ = 0.04 BF_10_ = 134.219									
	Group + Session: BF_10_ = 320.517											
Syllables reading	*F*(1.38, 53.82) = 7.38 *p*_[gg]_ = 0.004 η_p_^2^ = 0.16 BF_10_ = 12.03	*F*(1,39) = 0.006 *p* = 0.94 η_p_^2^ = 0.001 BF_10_ = 0.06	*F*(.38, 53.82) = 9.93 *p*_[gg]_ = < 0.001 η_p_^2^ = 0.20 BF_10_ = 1354.96	*t*(39) = 0.99 *p* = 0.33 *g* = 0.30 BF_10_ = 0.45	*t*(39) = − 0.23 *p* = 0.82 *g* = − 0.07 BF_10_ = 0.31	*t*(39) = − 0.57 *p* = 0.57 *g* = − 0.17 BF_10_ = 0.35	*p* > 0.008 *g* = 0.005 BF_10_ = 0.24	*p* > 0.008 *g* = 0.05 BF_10_ = 0.36	*p* > 0.008 *g* = 0.04 BF_10_ = 0.44	*t*(20) = − 3.14 *p* = 0.005 *g* = − 0.38 BF_10_ = 8.86	*t*(20) = − 3.43 *p* = 0.003 *g* = − 0.44 BF_10_ = 15.50	*t*(20) = − 1.01 *p* > 0.008 *g* = − 0.06 BF_10_ = 0.36
	Group + Session: BF_10_ = 7.47											
Word reading	*F*(1.08, 42.12) = 6.97 *p*_[gg]_ = 0.01, η_p_^2^ = 0.15 BF_10_ = 13.79	*F*(1,39) = 0.03 *p* = 0.87 η_p_^2^ = 0.001 BF_10_ = 0.72	*F*(1.08, 42.12) = 5.31 *p*_[gg]_ = 0.02 η_p_^2^ = 0.12 BF_10_ = 67.50	*t*(39) = 0.65 *p* = 0.52 *g* = 0.20 BF_10_ = 0.36	*t*(39) = − 0.01 *p* = 0.99 *g* = − 0.004 BF_10_ = 0.31	*t*(39) = − 0.14 *p* = 0.90 *g* = − 0.04 BF_10_ = 0.31	*p* > 0.008 *g* = − 0.03 BF_10_ = 0.49	*p* > 0.008 *g* = − 0.01 BF_10_ = 0.26	*p* > 0.008 *g* = 0.02 BF_10_ = 0.40	*p* > 0.008 *g* = − 0.23 BF_10_ = 2.95	*p* > 0.008 *g* = − 0.25 BF_10_ = 3.54	*p* > 0.008 *g* = − 0.02 BF_10_ = 0.58
	Group + Session: BF_10_ = 10.04											
Pseudoword reading	*F*(1.34, 52.26) = 9.95 *p*_[gg]_ = 0.001 η_p_^2^ = 0.20 BF_10_ = 101.18	*F*(1,39) = 0.04 *p* = 0.85 η_p_^2^ = 0.001 BF_10_ = 0.75	*F*(1.34, 52.26) = 5.48 *p*_[gg]_ = 0.01 η_p_^2^ = 0.12 BF_10_ = 573.88	*t*(39) = 0.62 *p* = 0.54 *g* = 0.19 BF_10_ = 0.36	*t*(39) = 0.10 *p* = 0.92 *g* = 0.03 BF_10_ = 0.31	*t*(39) = − 0.10 *p* = 0.92 *g* = − 0.03 BF_10_ = 0.31	*p* > 0.008 *g* = − 0.06 BF_10_ = 0.50	*p* > 0.008 *g* = − 0.02 BF_10_ = 0.25	*p* > 0.008 *g* = 0.04 BF_10_ = 0.36	*t*(20) = − 3.65 *p* = 0.002 *g* = − 0.20 BF_10_ = 24.22	*t(*20) = − 3.58 *p* = 0.002 *g* = − 0.22 BF_10_ = 21.07	*t*(20) = − 2.83 *p* > 0.008 *g* = − 0.03 BF_10_ = 4.89
	Group + Session: BF_10_ = 77.53											

*p*_*[gg]*_* p*-values with Greenhouse–Geisser adjustment, *BF*_*10*_ Bayes factor against the null model.

### BIN

**Figure 1:**
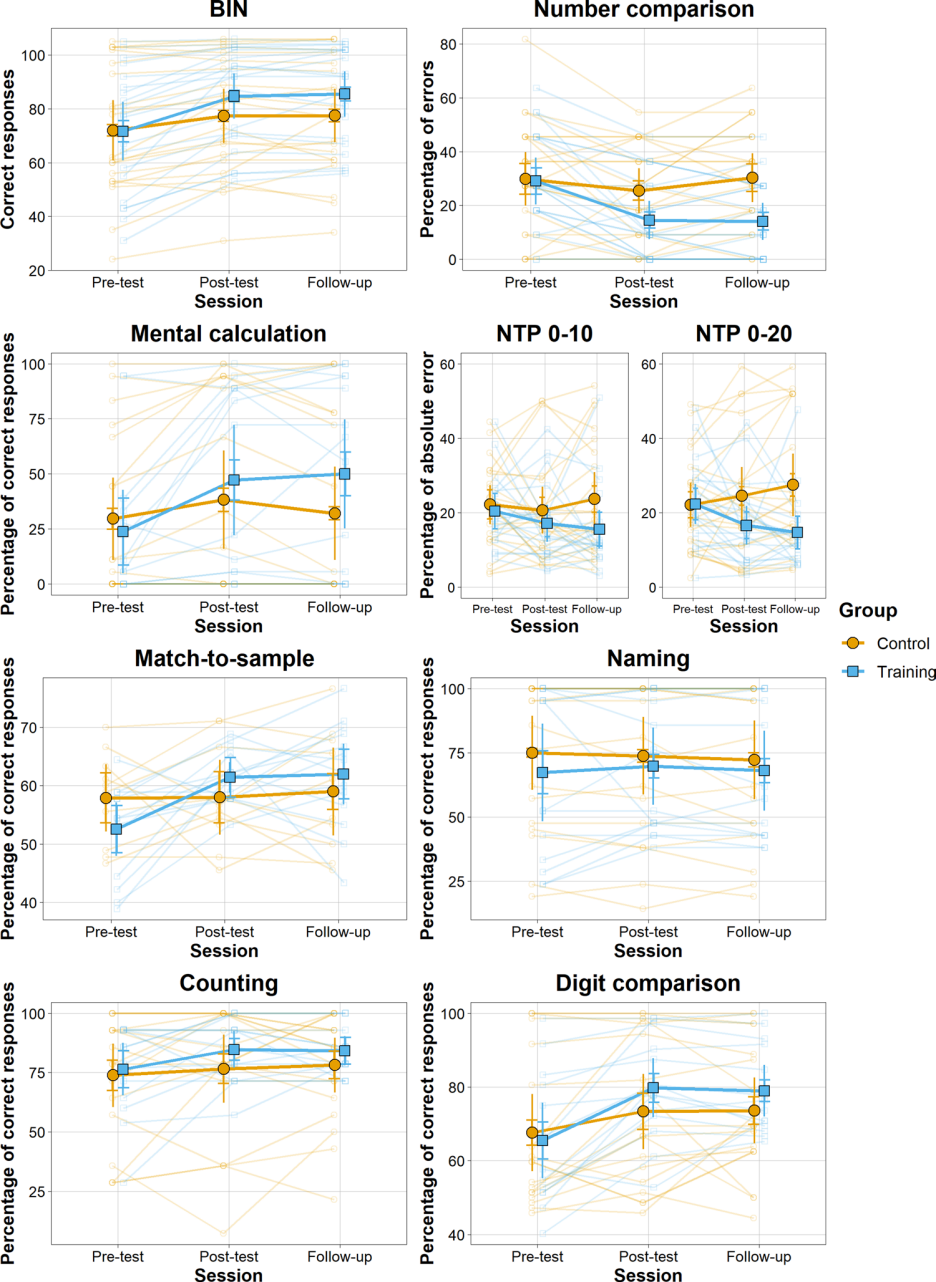
Mean scores in administered numerical tasks of EG and CG across sessions. Error bars represent between and within groups (horizontal segment) 95% confidence intervals. Transparent points represent individual scores.

We found strong evidence (BF = 19.46) in favour of the model with the two main effects and the interaction compared to the model with only the two main effects (Fig. 1). Although we found anecdotal evidence for a difference between the two groups at post-test and follow-up, both groups improved their scores from pre-test to post-test and from pre-test to follow-up but not from the post-test to follow-up. These improvements appear to be more substantial in the EG (extreme evidence) compared to the CG (strong evidence).

### Number comparison

**Figure 2:**
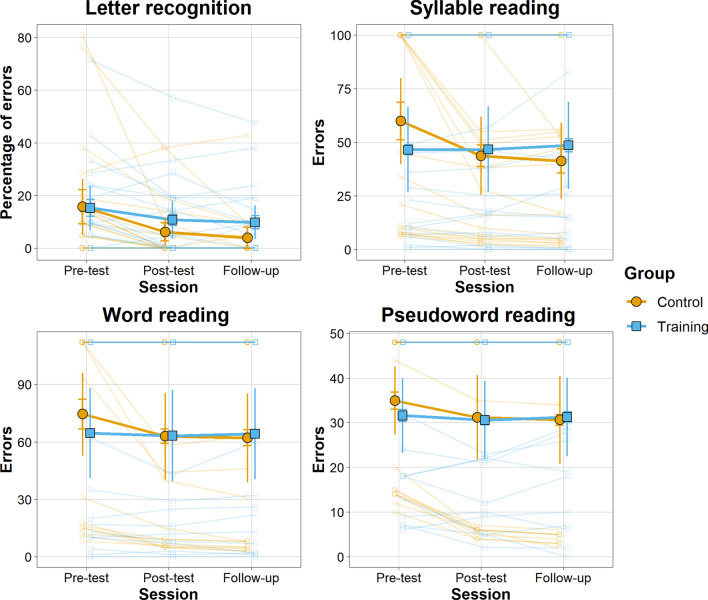
Mean scores in the administered literacy tasks for EG and CG across sessions. Error bars represent between and within groups (horizontal segment) 95% confidence intervals. Transparent points represent individual scores.

We found strong evidence (BF = 18.54) in favour of the model with the two main effects and the interaction compared to the model with only the two main effects (Fig. 2). The EG displayed a better performance compared to the CG at follow-up (moderate evidence). Moreover, only the EG reduced the errors from pre-test to post-test and such improvement was maintained until the follow-up (extreme evidence), which did not differ from the post-test.

### Mental calculation

We found moderate evidence (BF = 4.61) in favour of the model with the two main effects and the interaction compared to the model with only the two main effects (Fig. 1). Although we found anecdotal evidence for a difference between the two groups at post-test and follow-up, only the EG group displayed a better performance (moderate evidence) between pre-test and post-test and between pre-test and follow-up.

### Number to position task

For the 0–10, we found anecdotal evidence (BF = 0.405) in favour of the model with the two main effects compared with the model also including the interaction between session and group (Fig. 1). For the 0–20, there was moderate evidence (BF_10_ = 4.07) in favour of the model with the two main effects and the interaction and strong evidence (BF = 32.31) for its superiority compared to the model with only the two main effects (Fig. 1). The EG did not differ from the CG at pre-test, post-test, but did at follow-up (moderate evidence) by showing a better performance. However, we found anecdotal evidence for improvement between pre-test, post-test, and follow-up in the two groups.

### Match-to-sample

We found anecdotal evidence (BF = 2.59) in favour of the model with the two main effects and the interaction compared to the model with only the two main effects (Fig. 1). There was anecdotal evidence for a difference between groups across the three testing sessions. However, the EG displayed better performance in the post-test (strong evidence) and follow-up (moderate evidence) compared to the pre-test session.

### Number naming

We found moderate evidence in favour of the model with the main effects of session and group (BF = 0.22) compared to the model with additionally the interaction (Fig. 1).

### Counting

We found moderate evidence in favour of the model with the main effects of session and group (BF = 0.22) compared to the model with additionally the interaction (Fig. 1).

### Digit comparison

We did not find supporting evidence neither for the interaction between the two groups nor for the model with the two main effects only (BF = 1.01) (Fig. 1).

### Letter recognition

We found anecdotal evidence in favour of the model with the main effects of session and group (BF = 0.42) compared to the model with additionally the interaction (Fig. 2).

### Syllable reading

There was extreme evidence (BF = 181) in favour of the model with the two main effects and the interaction compared to the model with only the two main effects (Fig. 2). We found mainly anecdotal evidence for a difference between the two groups in the three testing sessions. However, there was moderate evidence for improvement between pre-test and post-test and pre-test and follow-up in the CG.

### Word and pseudoword reading

For word reading, there was moderate evidence (BF = 6.67) in favour of the model with the two main effects and the interaction compared to the model with only the two main effects (Fig. 2). There was mainly anecdotal evidence for a difference between the two groups in the three testing sessions. Only the CG group showed moderate evidence for improvement between pre-test and follow-up. For pseudoword reading, there was moderate evidence (BF = 7.42) in favour of the model with the two main effects and the interaction compared to the model with only the two main effects (Fig. 2). There was anecdotal evidence for a difference between groups in the testing sessions. However, the CG showed an improvement in performance (strong evidence) from pre-test to post-test and from post-test to follow up (moderate evidence).

## Discussion

Individuals with DS display numerical deficits that range from processing non-symbolic numerical quantities to arithmetic performance^12^. Several studies have evaluated interventions to improve numerical skills in DS yielding mixed results^71^. We provide new evidence to this research area by evaluating the efficacy of the computerized game “The Number Race” (a non-commercial, freely available software) in improving numerical skills in children with DS. We assigned participants to an experimental group (EG) who played with “The Number Race” and a control group (CG) who worked on reading skills using two different software. We evaluated participants’ numerical and reading skills before and immediately after the training as well as after three months. EG and the CG had similar numerical knowledge at pre-test and both groups displayed some improvement in their basic numerical skills immediately after the training, as measured by the numeracy battery (BIN). However, the EG showed a large improvement, whereas the CG obtained a small improvement. Note that the EG improvement on the numeracy battery (BIN), when evaluated against the normative data (which is available for preschoolers), corresponds to the (average) change observed between 51 and 73 months of age in typically developing children. Importantly, the level of basic numerical skills measured at the end of the training was maintained at the 3-months follow-up. The EG group displayed better performance in the number comparison task compared to the CG at follow-up, and only the EG showed such improvement from pre-test to post-test. The EG group also displayed improvements in mental calculation, which were maintained at follow-up, compared to their pre-test performance, even though there were no significant differences compared to the CG. The evidence for any changes in the CG’s scores in other numerical tasks was anecdotal at best. Conversely, the CG group showed a medium improvement in syllable reading and a small improvement in pseudoword reading at the end of the training. The latter score was further improved at follow-up in the CG. We found mainly anecdotal evidence for any improvement of reading skills in the EG.

Overall, we found substantial evidence for an improvement of the EG group in some but not all numerical tasks. We speculate that training had deeper effects on quantity understanding and manipulation, thereby yielding larger improvements in tasks that force children to actively manipulate numerical information (BIN, number comparison, and calculation) compared to others that do so to a lesser extent (e.g., number naming, counting). Nevertheless, the visual inspection of means suggests that the performance tended to increase across tasks. This suggests that twenty training sessions might not be enough to observe a substantial between-groups difference in children with DS, whereas they appear to be sufficient in typically developing children^73^. Future studies may explore whether longer training could make improvements in the numerical tasks more evident compared to the control group.

Another aspect worth considering is the large variability in children’s numerical and reading skills, which might have obscured differences between the two groups. Accordingly, some children might have found some aspects of the training superfluous, whereas others would have benefited more from practising on given components of the training. For example, the Number Race always requires players to move the characters on the board game aiming at improving the linear relation between space and numbers, which some participants may already possess, thereby making the training redundant. In this light, a training that aims at improving a variety of numerical skills at the same time, such as the Number Race, might dilute the capacity to generate a significant improvement in one specific skill in a limited amount of time. The Number Race cannot change its structure and fully adapt to the participant’s knowledge, which can instead be achieved in one-by-one training under the supervision of an expert. Sometimes what matters is not *what* is the training but *how much* it is trained. The moderate evidence for an improvement in mental calculation in DS^49^, which, instead, appeared to be stronger in typically developing preschool children^63^, might be due to the fact that the game requires to perform additions and subtractions only in the more advanced levels when participants accuracy in comparing symbolic numbers is maintained at a level of high accuracy^64^. Variable performance might have sent children with DS back to game levels that mainly involve number comparison, thereby preventing access to later game levels in which they could benefit from intense training on arithmetic. Not only *what* and *how much*, but also *how* a numerical skill is trained should be considered. In this vein, another possibility for the limited improvement in arithmetic is that the Number Race does not provide any support in terms of strategy. Admittedly, the Number Race presents the arithmetic operation in terms of dots that are added to or removed from a given set. However, there is no explicit instruction on using counting strategies such as counting-on from the larger set, which has been successfully taught to children with DS in a previous study^56^.

The CG showed some improvements in reading whereas the performance of the EG remained stable across reading tasks. Although the improvement in the CG might reflect a positive effect of the reading training, such evidence should be carefully considered. Accordingly, the CG tended to display lower error scores in the reading tasks that became more similar to those of the EG at post-test and follow-up. This amelioration might be due to simple regression to the mean, rather than a substantial effect of the reading training. The CG also displayed some amelioration in their numerical skills. However, the lack of a waiting list group prevents from disentangling whether this amelioration could be attributed to the simple effect of time or the effect of the reading training on early numerical skills^95^.

The Number Race simultaneously trains several numerical abilities such as non-symbolic and symbolic number comparison, counting, arithmetic, number-space association, and number recognition. In this light, the game enhances the link between different representations of numbers, which are the cornerstones of early numerical development^30,37^, while introducing the first arithmetical procedures. Nonetheless, it is difficult to draw strong theoretical conclusions on the relation between trained numerical skills, as done in other studies, whereby, for instance, training based exclusively on non-symbolic stimuli transferred to symbolic numbers^96,97^. In this regard, the Number Race aims at establishing and strengthening the main functional components of the cognitive architecture underlying number processing and mathematical learning^74^ rather than improving a specific numerical skill.

One theoretical conclusion concerns the trainability of numerical skills in DS. The descriptive statistics suggest that children with DS could improve in a variety of numerical skills, although the training might need to be longer and more intense compared to typical development. Yet, it is unclear whether individuals with DS have simply memorised some numerical facts and procedure (e.g., “seven is larger than six”, “one plus one equals two”), which still constitutes a valuable achievement, or have established a deep understanding of the numerical operations they have been practising. If the latter is the case, an improvement should be observed in numerical skills that were not targeted during the training (i.e., transfer). In this regard, the number line tasks could be considered as transfer tasks. Accordingly, children were not explicitly requested to place numbers onto a visual line during the game, although the number-space association was trained when children moved characters on the board. Children in the experimental group tended to show a more accurate placing of numbers on the line tasks, especially the 0–20 interval, compared to the control group at follow-up, thereby suggesting a real mastering of the numerical skills which were not directly trained. The same cannot be said for other tasks whose structure resembled the training proposed in the Number Race, such as comparing dots, as done in the match-to-sample task, or comparing symbolic numbers, as done in the number words and digits comparison tasks. Future studies should test whether training effects generalize to numerical activities that need to be carried out in daily life. The best way to put this hypothesis to test would be to obtain some real-life measures after the training to assess whether individuals with DS apply the learned numerical skills to different tasks and contexts. This would simultaneously assess the presence of a real transfer effect and the ecological validity of the training.

The conclusions of our study should be carefully considered in light of methodological limitations. The participants were alternately allocated to one of the two training groups with the exception of four participants who were allocated to the numerical training to meet the parents’ request. As a consequence, four participants were allocated to the reading training to balance the number of participants in each group. We decided to accommodate parents’ request to increase the compliance with the intervention, reduce attrition and increase our final sample size. The implemented assignment procedure, however, diverges from the standard random assignment, thereby requiring carefulness when interpreting the results. For instance, some children (and families) might have had a more positive attitude toward one of the interventions compared to the other. In this light, measuring expectations and attitudes toward the interventions can ensure that participants (and families) across groups have similar willingness to undergo the intervention^98^.

The small sample size may question the reliability of the findings. However, the pattern of results went in the expected direction with the EG improving in numerical tasks and the CG improving in reading tasks, also in the case of anecdotal evidence for a reliable improvement. Accordingly, a close look at the descriptive statistics reveals that the EG group displayed on average an increase in performance on almost all the administered numerical tasks, even though there was strong or extreme evidence only in a few instances. Larger sample sizes would provide more information on whether a given numerical skill can or cannot be effectively improved in the chosen time frame by training with the Number Race.

Another limitation is the lack of blinding regarding the training participants received. Research assistants conducting the assessment were aware of the group the participants belonged to. The lack of blinding might have generated a bias in the experimenters assessing the performance before and after the intervention. Future studies shall achieve blinding by having different experimenters for the supervision of the training and the assessment before and after the intervention.

A further limitation of our study is that we did not compare the computerized training with another numerical activity delivered in a more traditional way (e.g., one-to-one teaching with paper-and-pencil materials). Nevertheless, the use of an adaptive computerised task brings some intrinsic advantages as the game requires minimal supervision and can be easily implemented at home and school under the supervision of a non-expert (e.g., parent or teacher). Nonetheless, it remains an open question to identify the most beneficial training programme for DS not only in terms of increasing numerical and mathematical skills, but also in terms of cost–benefit for institutions, practitioners, families, and individuals.

Despite the above-mentioned limitations, the present study shows that The Number Race can be a promising tool to improve basic numerical skills in children with DS.

## Supplementary Information

Supplementary Information.

## Data Availability

The software “The Number Race” is freely available for non-commercial use at (http://www.thenumberrace.com/) in different language versions. All rights remain with their authors. The data that support the findings of this study are available from the corresponding author on reasonable request.
