# Supplementary material for: Training basic numerical skills in children with Down syndrome using the computerized game “The Number Race”
Source: Sci Rep. 2021 Jan 22;11:2087. doi: 10.1038/s41598-020-78801-5 (PMC7822821; doi:10.1038/s41598-020-78801-5)
Supplement: Supplementary file 1 — Supplementary Information. [file 41598_2020_78801_MOESM1_ESM.pdf]

Training basic numerical skills in children with Down syndrome using the computerized game “The Number Race”.

Francesco Sella[1], Sara Onnivello[2], Maristella Lunardon[2], Silvia Lanfranchi[2], and Marco Zorzi[2,3,*]

[1] Loughborough University, UK
[2] University of Padova, Italy
[3] IRCCS San Camillo Hospital, Venice-Lido, Italy

* correspondence: Prof. Marco Zorzi, Department of General Psychology and Padova Neuroscience Center, University of Padova, Italy, email: marco.zorzi@unipd.it

Supplementary Information

Variable	*M*	*SD*	1	2	3	4	5	6	7	8	9	10	11	12
1. BIN (correct responses)	71.85	23.66												
2. Number comparison (% of errors)	29.49	20.10	-.68** [-.82, -.47]											
3. Mental calculation (% correct)	27.08	35.10	-.09 [-.43, .26]	-.02 [-.37, .33]										
4. NTP 0-10 (PAE)	21.32	10.49	-.45** [-.66, -.16]	.54** [.28, .73]	.01 [-.34, .36]									
5. NTP 0-20 (PAE)	22.21	12.16	-.61** [-.78, -.38]	.48** [.20, .69]	-.11 [-.45, .24]	.70** [.50, .83]								
6. Match-to-sample (% correct)	54.77	8.29	.06 [-.35, .46]	-.17 [-.54, .25]	.00 [-.44, .45]	-.07 [-.46, .34]	-.23 [-.58, .19]							
7. Naming (% correct)	71.65	30.47	.13 [-.23, .46]	-.20 [-.51, .16]	.64** [.38, .81]	-.13 [-.45, .23]	-.12 [-.45, .24]	-.04 [-.48, .41]						
8. Counting (% correct)	74.69	21.36	.31* [.00, .57]	-.20 [-.48, .11]	-.01 [-.35, .34]	-.15 [-.44, .16]	-.31* [-.57, -.01]	.03 [-.38, .43]	.09 [-.26, .43]					
9. Digit comparison (% correct)	66.67	19.41	.05 [-.31, .39]	-.21 [-.52, .15]	.85** [.71, .92]	-.16 [-.48, .20]	-.16 [-.49, .19]	.01 [-.43, .45]	.71** [.49, .85]	.07 [-.28, .41]				
10. Letter recognition (% of errors)	15.45	20.70	-.68** [-.81, -.46]	.39* [.10, .62]	-.13 [-.46, .23]	.29 [-.02, .55]	.41** [.12, .64]	.01 [-.39, .41]	-.30 [-.59, .05]	-.29 [-.55, .02]	-.16 [-.49, .20]			
11. Syllable reading (errors)	53.34	43.29	-.81** [-.89, -.67]	.53** [.27, .72]	.01 [-.34, .36]	.38* [.08, .62]	.46** [.18, .67]	.05 [-.36, .44]	-.25 [-.55, .10]	-.47** [-.68, -.18]	-.11 [-.44, .25]	.56** [.30, .74]		
12. Word reading (errors)	69.66	48.56	-.80** [-.89, -.66]	.46** [.18, .68]	.02 [-.33, .36]	.33* [.02, .58]	.46** [.17, .67]	.09 [-.32, .48]	-.27 [-.57, .08]	-.33* [-.58, -.02]	-.07 [-.41, .29]	.55** [.29, .73]	.90** [.82, .95]	
13. Pseudoword reading (errors)	33.32	17.14	-.80** [-.89, -.65]	.45** [.16, .66]	-.04 [-.38, .32]	.29 [-.01, .55]	.44** [.15, .66]	.08 [-.33, .47]	-.28 [-.57, .08]	-.32* [-.57, -.02]	-.10 [-.43, .26]	.55** [.29, .73]	.89** [.80, .94]	.99** [.98, .99]

Table S1. *M* and *SD* are used to represent mean and standard deviation, respectively. Values in square brackets indicate the 95% confidence interval for each correlation. * indicates $p < .05$. ** indicates $p < .01$.
